# Characterization of cadmium accumulation mechanism between eggplant (*Solanum melongena* L.) cultivars

**DOI:** 10.3389/fpls.2022.1097998

**Published:** 2023-01-09

**Authors:** Chuang Shen, Ying-Ying Huang, Qiong Liao, Bai-Fei Huang, Jun-Liang Xin, Luo Wang, Hui-Ling Fu

**Affiliations:** Research Center for Environmental Pollution Control Technology, School of Chemical and Environmental Engineering, Hunan Institute of Technology, Hengyang, China

**Keywords:** eggplant, cadmium, root retention, translocation, cell wall

## Abstract

Excessive cadmium (Cd) accumulation in vegetables due to farmland pollution constitutes a serious threat to human health. Eggplant has a tendency to accumulate Cd. To investigate the mechanism of the differences in Cd accumulation levels between high-Cd (BXGZ) and low-Cd (MYQZ) eggplant cultivar, physiological and biochemical indicators and mRNA expression of eggplant were examined using photosynthetic apparatus, biochemical test kits, Fourier transform infrared (FTIR) spectroscopy and transcriptome sequencing, etc. The results of biochemical test kits and FTIR revealed that MYQZ enhanced pectin methylesterase (PME) activity, and lignin and pectin content in the root cell wall, which was associated with the upregulation of PME, cinnamyl-alcohol dehydrogenase and peroxidase (PODs). Higher levels of cysteine and glutathione (GSH) contents and upregulation of genes associated with sulfur metabolism, as well as higher expression of ATP-binding cassette transporters (ABCs), cation exchangers (CAX) and metal tolerance proteins (MTPs) were observed in MYQZ. In BXGZ, the higher stomatal density and stomatal aperture as well as higher levels of Ca^2+^ binding protein-1 (PCaP1) and aquaporins and lower levels of A2-type cyclins (CYCA2-1) are consistent with an enhanced transpiration rate in BXGZ. Furthermore, a more developed root system was shown to be associated with higher levels of auxin response factor (ARF19), GATA transcription factors (GATA4, 5 and 11) and auxin efflux carrier component (PIN5) in BXGZ. In conclusion, highly active PME, and higher levels of lignin and pectin in MYQZ are expected to reduce Cd toxicity, while Cd translocation can be inhibited with the help of ABC and other Cd transporters. As for BXGZ, the uptake and translocation of Cd were enhanced by the developed root system and stronger transpiration.

## 1 Introduction

Agricultural cadmium (Cd) pollution has posed a serious threat to farmland ecology and agricultural product safety due to its high biological toxicity and mobility (Kumar and Sharma, 2019). According to the survey of Li et al. (2018), 86% of paddy fields in the eastern Hunan area of China are subject to Cd contamination. Huang et al. (2017) reported that 70% to 90% of the accumulated Cd in humans is derived from the gastrointestinal intake of vegetables. Therefore, how to effectively reduce the accumulation of Cd in crops, inhibit the entry of Cd into the food chain, and ensure the safety of agricultural products has become a hot research topic among many interdisciplinary areas such as soil science, pollution ecology, environmental science and food science (Deng et al., 2020).

Eggplant (*Solanum melongena* L.) is a popular vegetable with high economic and nutritional value. According to the statistics collected by Agriculture Organization of the United Nations (FAO), China yielded 365,932,000 tons of eggplant in 2020 (FAOSTAT, 2020). However, eggplant has a tendency to accumulate excessive Cd, even when growing in lightly contaminated soils (Yuan et al., 2019). To control the Cd accumulation level of eggplant in a safe range through pollution-safe cultivar (PSC) strategy, a low-Cd cultivar (MYQZ) and a high-Cd cultivar (BXGZ) were obtained in our previous study (Shen et al., 2021). The low-Cd cultivar showed high Cd retention and sequestration ability in the root, leading to reduced Cd transportation from roots to fruits in eggplants. While the relevant molecular mechanisms are yet to be not well established.

The main stages of Cd uptake and its movement have involved root uptake, xylem loading and translocation to shoots (Uraguchi and Fujiwara, 2013). The lower Cd concentrations in the seeds of *Triticum aestivum* and *Ricinus communis* and shoot of *Solanum melongena* cultivars may be related to their lower root uptake capacity of Cd, but also to the relatively low capacity of Cd translocation through xylem and phloem (Qin et al., 2015; Ye et al., 2018). The molecular mechanism of Cd uptake and transport in plants has been relatively well investigated. The iron-regulated transporter 1 (IRT1) the principal root transporter for Cd uptake from the soil, and plants overexpressed IRT1 inclined to accumulate more Cd than wild-type plants (Barberon et al., 2014). Natural resistance-associated macrophage protein (NRAMP5) is also a key Cd transporter contributing to Cd uptake, and knockdown of OsNramp5 has been employed to obtain rice cultivars with low Cd accumulation (Tang et al., 2017; Ismael et al., 2019). *OsHMA3* regulates Cd sequestration in the vacuole of roots, and the overexpression of *OsHMA3* of rice showed that Cd accumulation was considerably elevated in roots and intensely decreased in shoots (Miyadate et al., 2011; Sasaki et al., 2014). Transporters such as ABCs (ATP-binding cassette transporters) and CAXs (cation exchanger) that localize to the vacuolar membrane are responsible for Cd sequestration in root vacuoles and the reduction of Cd translocation and accumulation in the shoots (Shahid et al., 2017; Huang et al., 2020).

Transpiration pull is an important force for Cd translocation from roots to shoots. It has been found that plants modulate transpiration by controlling root water uptake (Cai et al., 2022) and regulating stomatal density and closure under Cd stress (Agurla et al., 2018; Tao et al., 2021). Similar studies between high- and low-Cd cultivars of pakchoi, wheat and sweet potato demonstrated that longer root, more root tips, larger root surface and volume contributed to higher Cd uptake and translocation (Kubo et al., 2011; Xia et al., 2016; Xin et al., 2017). Furthermore, sulfur metabolism-related products such as phytochelatins (PCs), glutathione (GSH), and metallothionein (MT) are able to chelate free Cd ions in plants through the sulfhydryl groups, an important process for the detoxification and accumulation of Cd in plants (Gill and Tuteja, 2011; Yang et al., 2021). Cell wall components and the degree of methylation of the cell wall determine the ability of the root cell wall to adsorb Cd, and ultimately influences the translocation of Cd to shoot (Zhu et al., 2012). Pectin, cellulose, hemicellulose and lignin, etc., are important components of the root cell wall, containing many functional groups (-COOH, -OH, -SH, etc.), which help to adsorb and fix Cd ions and reduce its entry into the protoplasts, thus greatly reducing the toxicity of Cd suffered by plants (Loix et al., 2017; Wu et al., 2020). Nevertheless, studies on the physiological, biochemical and molecular mechanisms of low Cd accumulation in crops have mainly emphasized on grain crops, while relatively few researches have been conducted on vegetables, especially eggplant.

Therefore, based on the low-Cd and high-Cd eggplant cultivars identified in our previous study, the mechanism of Cd uptake, transport and accumulation of eggplant at the physiological, biochemical and genetic levels are explored in the present study. We hypothesized that (i) the differences in the expression of genes involved in Cd uptake and transport contribute to the variation in Cd accumulation between low-Cd and high-Cd eggplant cultivars; (ii) physiological and biochemical distinctions related to Cd stress between low-Cd and high-Cd eggplant cultivars could also be the primary factor responsible for the differences in Cd accumulation capacity. The results of this study are expected to shed light on the mechanisms of Cd accumulation in eggplant, as well as to facilitate the screening and breeding of Cd-PSC vegetables.

## 2 Materials and methods

### 2.1 Plant materials and experimental design

The high-Cd cultivar (BXGZ) and low-Cd cultivar (MYQZ) of eggplant used in this study were obtained from a previous screening trial. Eggplant seeds of BXGZ and MYQZ were surface sterilized with 5% hydrogen peroxide and sown in a Petri dish for germination. Seedlings were transferred to sand and grew for one week after germination. Then eggplant seedlings with consistent growth condition were selected and planted in 500 ml cups for hydroponics with half-strength Hoagland solution. Seedlings were grown in a plant culture chamber at of 28°C and 250 μmol/m^2^/s light intensity (14 h per day), and the half-strength Hoagland solutions were changed every 3 days to ensure optimal growth conditions for eggplants. After 10 days of growth, seedlings of each eggplant cultivar were randomly divided into 2 groups, one was treated with Cd-free half-strength Hoagland solutions as control (CK), and the other was treated with Cd concentration of 10μM half-strength Hoagland solutions as an experimental group (Cd). The samples of eggplant seedlings were harvested after 2 weeks of culture and 3 seedlings were used as biological replicates for each treatment.

### 2.2 RNA sequencing, analysis and validation

Total RNAs of eggplant root samples were isolated through EASYspin Plant RNA kit (Aidlab, China). Four cDNA libraries with 3 biological replicates each were constructed from reverse transcription of total RNA and sequenced with the Illumina HiSeq2500 (Illumina, CA, USA). The raw data have been uploaded in the SRA database of NCBI, and the BioProject accession number is PRJNA908752. To ensure high quality sequencing, the raw reads were processed to clean reads using fastp 0.18.0. Gene expression levels for each cDNA library were normalized by fragment per kilobase of transcript per million mapped reads (FPKM). Differentially expressed genes (DEGs) were identified by the criteria of false discovery rate (FDR) < 0.05 and |log_2_ fold-change value| ≥1. Gene Ontology (GO) and KEGG pathway analyses of the DEGs were accomplished through Blast2GO 2.8 and BLASTX.

RNA-Seq results were verified by quantitative real-time PCR (qPCR). The cDNA templates used for qPCR were obtained through the reverse transcription of the remaining total RNA with the help of PrimeScript™ RT Master Mix kit (Takara, Japan). Five DEGs were randomly selected for qPCR experiments on CFX96 Touch instrument (Bio-Rad Laboratories, Hercules, CA) coupled with SYBR Green II PCR Master Mix kit (Takara, Japan), and the qPCR results were calculated using the _ΔΔ_Ct method (Schmittgen and Livak, 2008).

### 2.3 Determination of Cd concentrations, pectin methylases and peroxidase activity, cysteine synthase, glutathione and oxidized glutathione concentrations

Samples of 0.1 g of oven-dried plants were fully digested in a microwave oven (Microwave Digester XT-9900A, Shanghai Xintuo Analytical Instruments Co., Ltd., China) with HNO_3_:H_2_O_2_ (10:3, v/v). The digested samples were fixed to 15 ml with 1% nitric acid, and then Cd concentrations were measured by flame atomic absorption spectrophotometer (Hitachi Z-2300, Japan). To ensure data accuracy and quality control of the test, plant GBW07605 (provided by the National Research Center for CRM, China) was used as the certified reference material with a recovery rate of 97.21% and a relative standard deviation of 3.2% for Cd.

The fresh root samples of BXGZ and MYQZ treated with the CK and Cd were used for this experiment. The pectin methylesterase (PME) activity, cysteine synthase, glutathione (GSH), oxidized glutathione (GSSG) and peroxidase (POD) activity were tested according to the instructions of PME, cysteine synthase, GSH, GSSG and POD assay kits from Suzhou Comin Biotechnology (China), respectively.

### 2.4 Measurement of transpiration rate and stomatal characteristics

The transpiration rate of eggplant was measured using the portable photosynthetic apparatus HM-GH60 (Hengmei Electronic Technology Co., Ltd, China). The epidermal cells of the eggplant leaves were carefully excised and photographed by an optical microscope (Olympus BX53, Olympus Corporation, Japan). The stomatal density was analyzed *via* the number of stomata divided by the leaf area. All the tested leaves were picked from the same position of three different eggplant seedlings under the same light condition.

### 2.5 Determination of root cell wall lignin contents and characteristics

The lignin extraction of eggplant root cell wall was performed according to the method of Gao et al. (2021). Fresh samples of 2g of roots were well ground in liquid nitrogen and then thoroughly mixed with 20 ml of 75% ethanol. After standing in an ice bath for 20 min, the root samples were centrifuged at 8000g for 10 min at 4°C. The collected precipitates were sequentially washed with 14 ml of acetone, methanol/chloroform (1:1) and methanol each for 10 min, and then centrifuged at 5000g for 10 min at 4°C to refine the precipitates. The refined precipitates were air-dried at 40°C to obtain root cell walls and then stored at 4°C for further use. Thoroughly grind 1 μg of root cell walls and 150 mg of potassium bromide (KBr) with an agate mortar and then press into thin slices. The thin slices were placed into a Fourier transform infrared (FTIR) spectroscopy (Nicolet iS20, Thermo Fisher, USA) to identify the absorbance values of the functional groups of the root cell wall in the wavelength range of 400-4000 cm^-1^ with a resolution of 4 cm^-1^.

### 2.6 Root morphological analysis

Root systems of BXGZ and MYQZ were scanned with Epson Perfection V700 Photo (Seiko Epson Corporation, Japan). The data of the root surface area and tip number were identified on scanned images of BXGZ and MYQZ with the help of root image analysis software Win-RHIZO Pro (Regent Instruments, Canada). Three roots of BXGZ and MYQZ under each treatment were randomly selected for replication.

### 2.7 Statistical analysis

Both SPSS 23.0 (IBM Inc., USA) and Excel 2016 (Professional Edition, Microsoft, USA) were used to perform statistical analysis and figure development. Two-way analysis of variance (ANOVA) and least significant difference (LSD) tests were employed to determine the significance of data. Results were considered statistically significant when *p*<0.05.

## 3 Results

### 3.1 Biomass and cadmium concentration of eggplants

**Figure 1:**
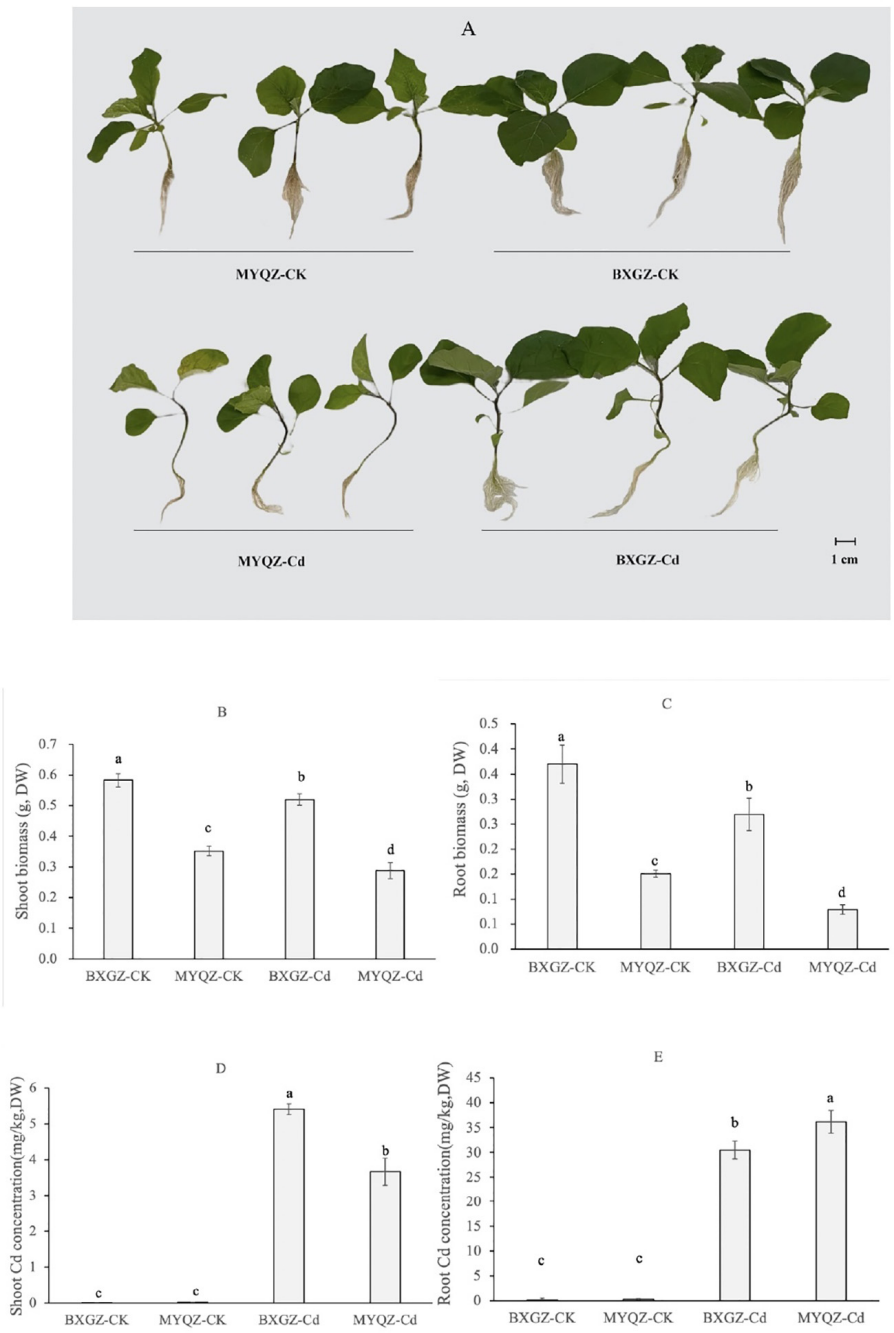
The growth status **(A)**, shoot biomass **(B)**, root biomass **(C)**, the shoot and root Cd concentrations **(D, E)** eggplants exposed to 10 μM Cd for 2 weeks. Values are the mean ± standard error (n = 3). Different small letters indicate significant differences (*p* < 0.05) among the four treatments.

The results of two-way ANOVA showed that shoot and root biomasses (dry weight, DW) were significantly affected by Cd treatment and cultivar (*p*<0.01), but not by treatment×cultivar (*p*>0.05) (**Table S1, S2**, supporting information), indicating that the shoot and root biomasses eggplant were determined by both Cd treatment and cultivar. BXGZ and MYQZ were in good growth status under CK treatment, and both shoot and root biomass of BXGZ were significantly higher than those of MYQZ (*p*<0.05) (**Figures 1A-C**). Under Cd treatment, the shoot and root biomass of BXGZ decreased by 10.7% and 27.1%, respectively, while a 18.1% and 47.6% reduction of shoot and root biomass were observed in MYQZ, in comparison to the CK treatment (**Figures 1A-C**). Despite the significant reduction in biomass in both BXGZ and MYQZ, the reduction was more pronounced in the root system, and biomass reduction in BXGZ was significantly lower than that in MYQZ (*p*<0.05), indicating that Cd treatment exerts a greater impact on the root and BXGZ exhibited a stronger Cd tolerance in comparison to MYQZ.

To verify the stability of Cd accumulation of eggplant, Cd concentrations were also measured. Two-way ANOVA revealed that shoot and root Cd concentrations (DW) were significantly affected by Cd treatment and cultivar (*p*<0.01), as well as by treatment×cultivar (*p*<0.01) (**Table S3, S4**, supporting information). The Cd accumulation levels of BXGZ and MYQZ were consistent with the results of our previous soil experiments. The shoot Cd concentration of MYQZ was significantly lower than that of BXGZ (*p*<0.05), while the root Cd concentration of MYQZ was significantly higher than that of BXGZ (*p*<0.05), indicating that BXGZ and MYQZ were stable in Cd accumulation characteristics and significant differences existed between them for Cd uptake and translocation (**Figure 1D**).

### 3.2 Overview of the RNA-sequencing results

To investigate the variation of molecular mechanisms between BXGZ and MYQZ in response to Cd stress, the differences in the quantity and function of DEGs between BXGZ and MYQZ under CK and Cd treatments were comparatively analyzed. A total of 1183 up-regulated and 717 down-regulated DEGs were identified in BXGZ compared to MYQZ under CK treatment (BXGZ-CK vs MYQZ-CK), and 1918 up-regulated and 1267 down-regulated DEGs were observed in BXGZ compared to MYQZ under Cd treatment (BXGZ-Cd vs MYQZ-Cd). In the comparative analysis of BXGZ-Ck vs BXGZ-Cd and MYQZ-Ck vs MYQZ-Cd, the number of both up-regulated and down-regulated DEGs was also higher in BXGZ than in MYQZ, indicating that there were significant genotypic differences between BXGZ and MYQZ, as well as BXGZ were more responsive to Cd stress (**Figure S1**). The qPCR result of five randomly selected genes was consistent with the transcriptome profiles, indicating the credibility of the RNA-sequencing results (**Figure S2**).

**Figure 2:**
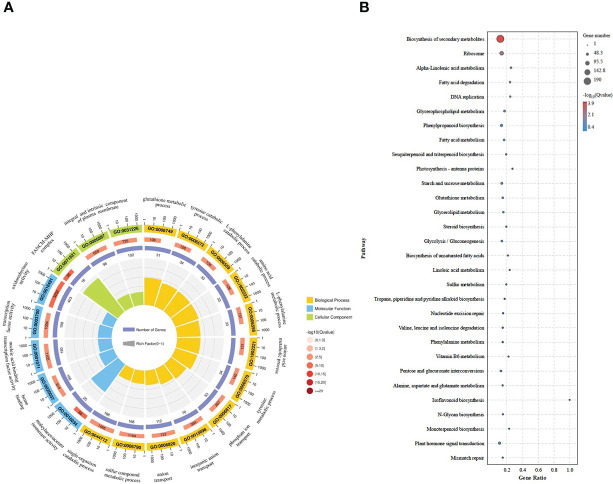
The GO **(A)** and KEGG **(B)** analysis of the DEGs in BXGZ-Cd vs MYQZ-Cd.

To elucidate the specific functions of the DEGs obtained in BXGZ-Cd vs MYQZ-Cd, Gene Ontology (GO) and Kyoto Encyclopedia of Genes and Genomes (KEGG) enrichment analyses were performed. The majority of DEGs were enriched in the GO term of oxidoreductase activity (GO:0016491), nucleic acid binding transcription factor activity (GO:0001071), component of plasma membrane (GO:0005887 and 0031226), L-phenylalanine catabolic and metabolic process (GO:0006559 and GO:0006558) and sulfur compound metabolic process (GO:0006790), etc. (**Figure 2**). For the KEGG pathways, the biosynthesis of secondary metabolites (such as phenolic and flavonoid biosynthesis), phenylpropanoid biosynthesis, glutathione metabolism, cysteine and methionine metabolism, plant hormone signal transduction and sulfur metabolism were enriched (**Figure 2**). Most of these enriched GO and KEGG terms are associated with plant growth and stress resistance (Huang et al., 2022). Therefore, the enrichment analysis of GO and KEGG suggested that BXGZ and MYQZ have different strategies to cope with Cd stress, mainly focusing on antioxidant, sulfur metabolism, and phenylalanine synthesis, etc.

### 3.3 The DEGs involved in transpiration, root development, cell wall components, sulfur metabolism and cadmium transporters

**Figure 3:**
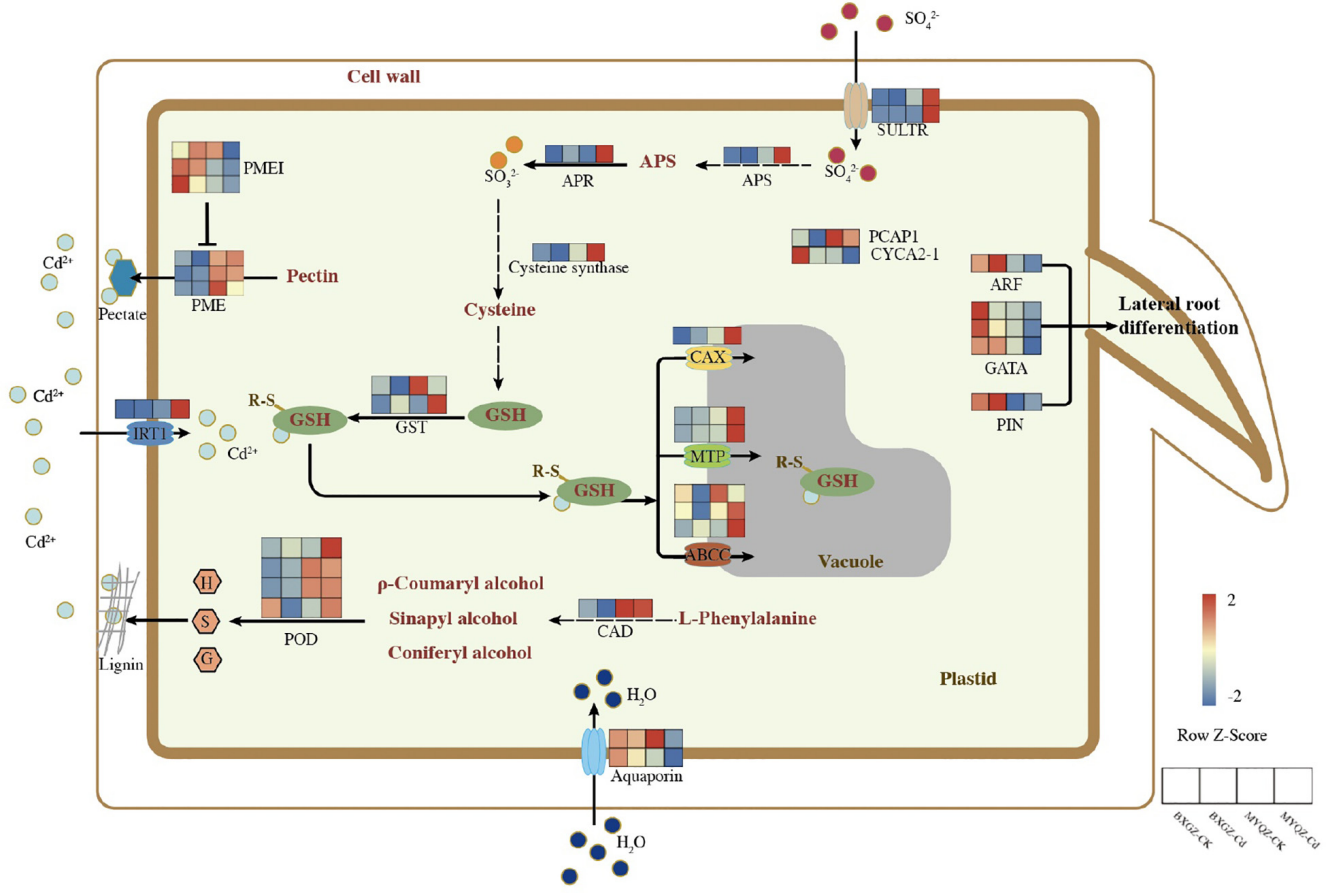
The schematic diagram of DEG in response to Cd stress between different treatments in the roots of BXGZ and MYQZ. The expression heatmap was arranged as BXGZ-CK, BXGZ-Cd, MYQZ-CK, and MYQZ-Cd from left to right. The expression level of each DEG was averaged by the 3 biological samples and then normalized to a z-score.

Based on the results of DEGs, the schematic diagram of DEGs in response to Cd stress were summarized (**Figure 3**). Two genes related to stomatal development and closure were identified, namely Ca^2+^-binding protein-1 (*PCaP1*) and A2-type cyclins (*CYCA2-1*). *PCaP1* was identified to trigger stomatal closure by involving in Ca^2+^ signaling, and the overexpression of *CYCA2-1* was observed to result in reduced stomatal density (Nagata et al., 2016; Qu et al., 2018). The expression level of *PCaP1* in MYQZ was significantly higher than that in BXGZ, while *CYCA2-1* showed the opposite (**Figure 3**). Aquaporins has been confirmed to enhance transpiration by regulating the water uptake in roots (Sinclair et al., 2017). The expression level of *aquaporins* in BXGZ roots was also significantly higher than that in MYQZ (*p*<0.05) when subjected to Cd stress (**Figure 3**).

The DEGs related to lateral root development such as *auxin response factor* (*ARF19*), *GATA transcription factors* (*GATA4, 5 and 11*) and *auxin efflux carrier component* (*PIN5*) were shown significantly higher levels in BXGZ than in MYQZ (*p*<0.05) under either CK or Cd treatment, and the expression of these genes was significantly down-regulated in both cultivars under Cd treatment (**Figure 3**).

The expression levels of *cinnamyl-alcohol dehydrogenase* (*CAD14*) and *PODs* (*PER3, 52, 53* and *72*) related to lignin synthesis were significantly higher in MYQZ than in BXGZ (*p*<0.05) (**Figure 3**). In addition, the expression levels of pectin related genes such as *pectin methylesterase* (*PME 26, 40* and *46*) were significantly higher (*p*<0.05) and *PME inhibitor* (*PMEI 7* and *9*) were significantly lower (*p*<0.05) in MYQZ than BXGZ (**Figure 3**), respectively.

The differences in the expression of sulfur metabolism-related genes between MYQZ and BXGZ under CK treatment were not significant (*p*>0.05) or slightly higher in MYQZ than in BXGZ (**Figure 3**). However, under Cd stress, the expression levels of genes concerned with *sulfate transporters* (*SULTR1;3 and SULTR4;2*), *ATP sulfurylase* (*APS1*), *adenylylsulfate reductase* (*APR2*), *cysteine synthase* (*RCS3*) and *glutathione S-transferase* (*GST1 and 3*) were significantly higher in MYQZ than in BXGZ (*p*<0.05), respectively, indicating a more intensive level of sulfur metabolism in MYQZ under Cd treatment.

Four types of Cd transport-related membrane proteins were identified. Among them, the Cd transporter located on the cell membrane was iron-regulated transporter 1 (IRT1), which serves to enhance the Cd uptake by roots (Barberon et al., 2014). The expression of *IRT1* was relatively low in both MYQZ and BXGZ under CK treatment but was significantly elevated (*p*<0.05) only in MYQZ under Cd treatment. The other three types of Cd transporters were all located on the vacuole, namely ATP-binding cassette transporters (*ABCC4, 9 and 17*), cation exchanger (*CAX5*) and metal tolerance protein (*MTP4 and 10*), and these three transporters are mainly in charge of transporting Cd from the cytoplasm to the vacuole where it can be compartmentalized (Huang et al., 2020). In terms of overall expression levels, these three types of Cd transporters were not significantly (*p*>0.05) different under CK treatment, while they were significantly higher (*p*<0.05) in the roots of MYQZ than BXGZ under Cd treatment (**Figure 3**).

### 3.4 Stomatal characteristics and transpiration rate under Cd treatment

**Figure 4:**
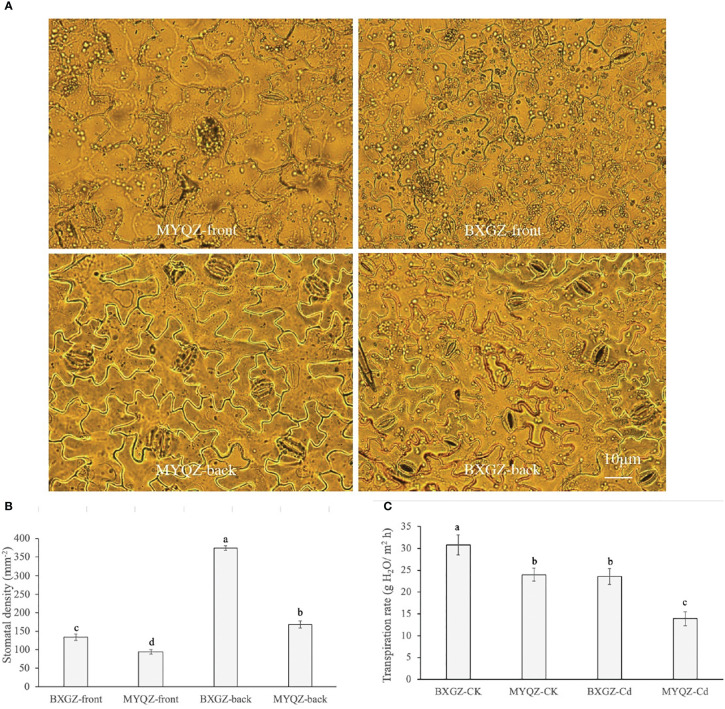
The micrographs of front and back epidermis **(A)**, stomatal density **(B)**, and transpiration rate **(C)** of BXGZ and MYQZ. Values are the mean ± standard error (n = 3). Different small letters indicate significant differences (*p* < 0.05) among the four treatments.

The stomatal distribution per unit area of eggplant leaves on both the front and back sides under Cd treatment was shown in **Figure 4**. The density of stomata on the front sides of both BXGZ and MYQZ leaves was significantly lower than that on the back sides, and the density of stomata of BXGZ was significantly higher than that of MYQZ (*p*<0.05) (**Figure 4**). Accordingly, the differences in expression levels of *PCaP1*and *CYCA2-1* in MYQZ and BXGZ were consistent with our microscopic observations.

The transpiration rate was significantly affected by Cd treatment and cultivar (*p*<0.01), but not by treatment × cultivar (*p*>0.05) (**Table S5**, supporting information). The transpiration rate was positively correlated with the density of stomata and the expression level of *aquaporins* in eggplant, which was significantly higher in BXGZ than in MYQZ under Cd stress (*p*<0.05) (**Figure 4**), suggesting that BXGZ showed stronger transpiration rate under Cd stress.

### 3.5 Root morphology and development

**Figure 5:**
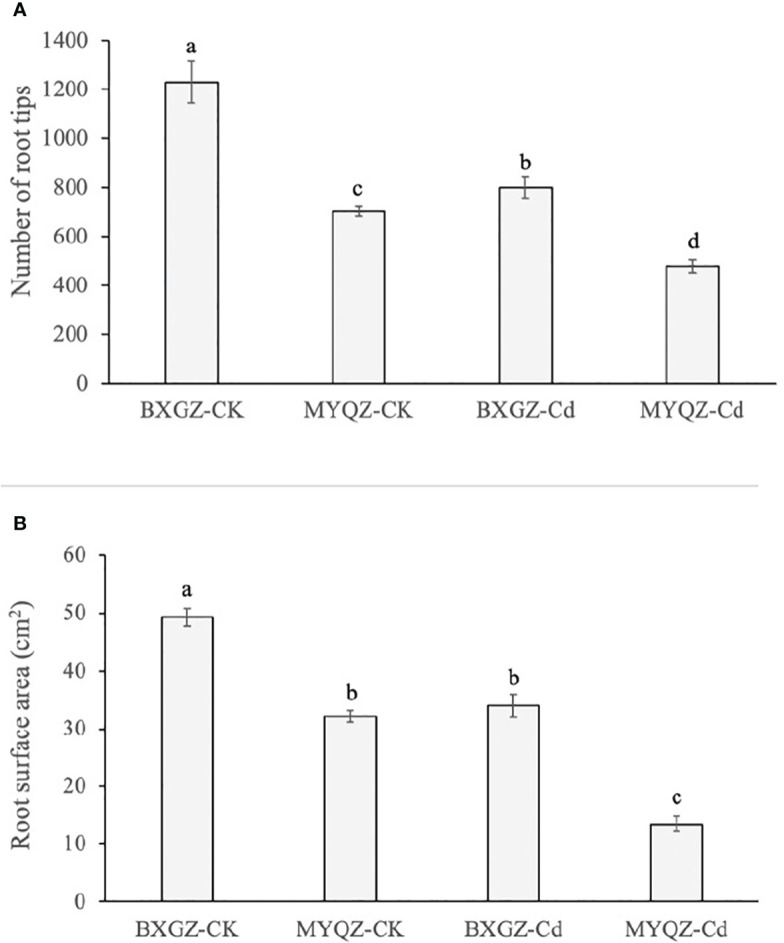
The root tips **(A)** and surface area of **(B)** BXGZ and MYQZ under different treatments. Values are the mean ± standard error (n = 3). Different small letters indicate significant differences (*p* < 0.05) among the four treatments.

BXGZ showed more developed lateral roots than MYQZ, and both the root development of BXGZ and MYQZ was affected by Cd stress (**Figure 1**). The number of root tips was affected by Cd treatment, cultivar and treatment × cultivar (*p*<0.01) (**Table S6**, supporting information), while the root surface area was only affected by Cd treatment and cultivar (*p*<0.01) (**Table S7**, supporting information). Under Cd stress, the root tips and surface area of BXGZ were significantly higher than those of MYQZ (*p*<0.05), and its root tips and surface area decreased at a significantly lower level than those of MYQZ (*p*<0.05) (**Figure 5A, B**). Although Cd stress affected eggplant root development, in terms of root tip number and root surface area, the roots of BXGZ were significantly less affected compared to MYQZ, which may be attributed to the difference in Cd tolerance between the cultivars

### 3.6 Lignin, PME and functional groups of root cell walls

**Figure 6:**
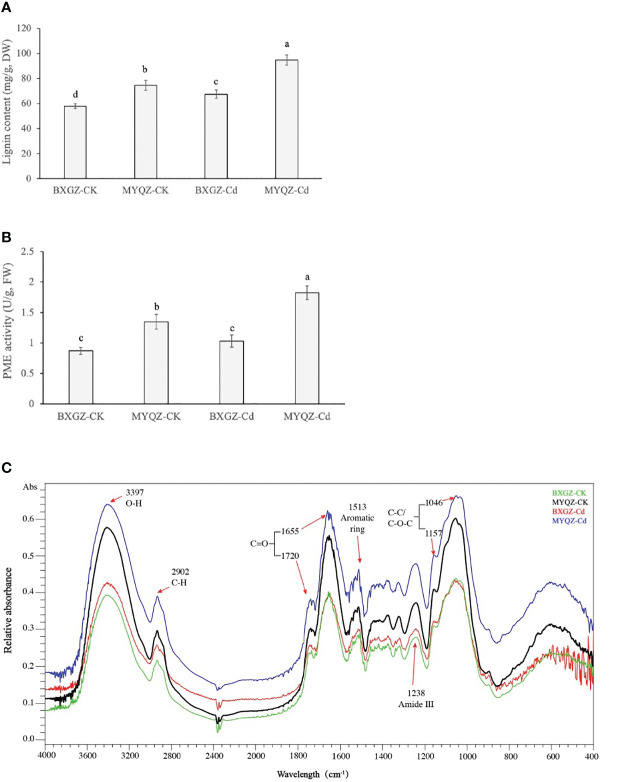
The lignin contents **(A)**, PME activities **(B)** and FTIR spectral analysis **(C)** of BXGZ and MYQZ under different treatments. Values are the mean ± standard error (n = 3). Different small letters indicate significant differences (*p* < 0.05) among the four treatments.

Lignin is produced in a combination of three lignin monomers (H-, G-, and S-lignin) through the processing of phenylalanine catalyzed by multiple enzymes. The lignin content of eggplant roots was affected by Cd treatment, cultivar (*p*<0.01) and treatment × cultivar (*p*<0.05) (**Table S8**, supporting information). The results of lignin content of eggplant roots were in agreement with the expression levels of genes related to lignin synthesis as well, which were significantly higher in MYQZ than in BXGZ (*p*<0.05) (**Figures 6**). The results of two-way ANOVA showed that the PME activity in eggplant roots was affected by Cd treatment, cultivar (*p*<0.01) and treatment × cultivar (*p*<0.05) (**Table S9**, supporting information). As shown in **Figure 6**, PME activity in MYQZ was significantly higher than that in BXGZ under both CK and Cd treatments (*p*<0.05). PME activity in BXGZ showed almost no effect under Cd stress (*p*>0.05), while its activity in MYQZ was significantly elevated after Cd treatment (*p*<0.05).

The characteristics of the functional groups of root cell walls were determined by the absorbance values at different wavelengths by FTIR spectroscopy. As shown in **Figure 6**, the FTIR wave peaks of eggplant root cell walls were most significant at 3397, 1655 and 1046 cm^-1^, representing the O-H of polysaccharides (Regvar et al., 2013), C=O of protein and C-C or C-O of pectin and polysaccharides respectively (Gao et al., 2021). In addition, the elevated absorbances were observed at 2902, 1720, 1513, 1238 and 1157 cm^-1^, which were assigned to the C-H of cellulose and pectin, C=O of methyl-esterified pectin, aromatic ring of lignin, amide III and C-C or C-O of lignin and polysaccharides respectively (Regvar et al., 2013; Lahlali et al., 2017). In terms of relative absorbance values, the content of functional groups associated with pectin and lignin was significantly higher in MYQZ cell walls than in BXGZ (*p*<0.05) under either CK or Cd treatment. Moreover, Cd stress led to a significant elevation in the content of relevant groups in the cell wall of MYQZ, while these groups of BXGZ was only slightly increased at 3397, 2902 and 1238 cm^-1^ (**Figure 6**).

### 3.7 Indicators involved in sulfur metabolism and POD activities

**Figure 7:**
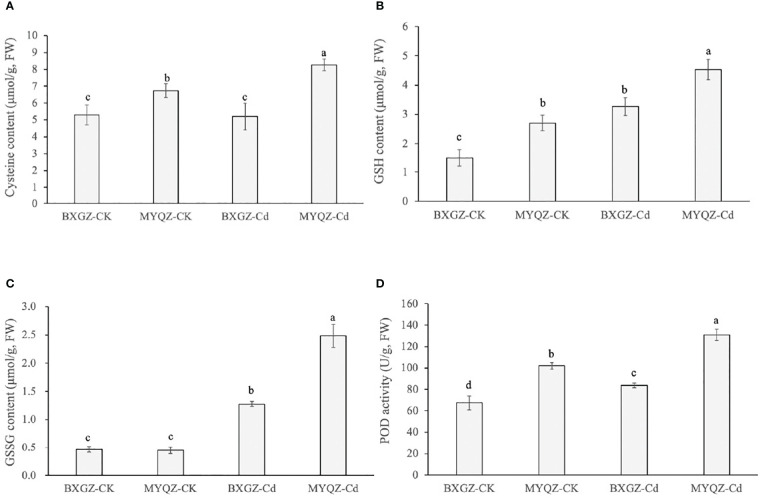
The cysteine contents **(A)**, GSH contents **(B)**, GSSG contents **(C)** and POD activities **(D)** of BXGZ and MYQZ under different treatments. Values are the mean ± standard error (n = 3). Different small letters indicate significant differences (*p* < 0.05) among the four treatments.

The sulfur metabolism pathway is an important contributor of thiol groups (-SH) in plants, which can chelate with Cd ions and reduce the toxicity of Cd to plants. The results of two-way ANOVA showed that cysteine content was significantly affected by cultivar (*p*<0.01) and treatment × cultivar (*p*<0.05) (**Table S10**, supporting information). The GSH content was only affected by both Cd treatment and cultivar (*p*<0.01) (**Table S11**, supporting information). As for GSSG and POD contents, they were significantly affected by Cd treatment, cultivar and treatment × cultivar (**Table S12, S13**, supporting information). In addition, the content of cysteine in the roots of MYQZ was significantly increased (*p*<0.05) and significantly higher than that of BXGZ (*p*<0.05) (**Figure 7**). The contents of GSH and GSSG in BXGZ and MYQZ were also significantly increased under Cd stress (*p*<0.05), but the content in MYQZ was remarkably higher than BXGZ (*p*<0.05) (**Figure 7B**). The content of peroxidase (POD) was consistent with the expression level of *POD* (*PER3, 52, 53 and 72*), both of which exhibited significantly higher in MYQZ than in BXGZ under Cd stress (*p*<0.05) (**Figure 7**).

## 4 Discussion

As an important vegetable, the investigation of Cd accumulation characteristics of eggplant is of great importance. The root and shoot Cd concentrations of BXGZ and MYQZ were consistent with previous studies, suggesting the Cd accumulation capacity of eggplant should be cultivar-dependent. Studies have shown that the growth dilution (same uptake but larger biomass) contributes to the reduction of Cd concentration (Shi et al., 2016; Ismael et al., 2019). However, the biomass of BXGZ was significantly higher than that of MYQZ under both CK and Cd treatments. According to Dai et al. (2021), there was no significant correlation between the Cd content of plants and their biomass, instead it was mainly affected by the root uptake and fixation, transpiration and Cd transporters. To investigate the mechanisms underlying the differences in Cd accumulation capacity of eggplant, the molecular and physiological mechanisms of BYQZ and MYQZ, which are closely related to Cd accumulation, were investigated in this study.

### 4.1 Root cell wall formation contributed to cadmium fixation in eggplant

The root cell wall is an important organ for plants to achieve self-protection against environmental stresses (Guo et al., 2021). Increased content of cell wall components bound to Cd ions has been observed in plants such as *Oryza sativa* L. (rice), *Zea mays* L. (maize), as an effective strategy for plants to cope with Cd stress (Vatehová-Vivodová et al., 2018; Riaz et al., 2021). The stronger the root cell wall fixation of Cd, the lower the amount of Cd that can enter the root cells, and the less can be transported to the shoot and fruit (Huybrechts et al., 2019).

Among the components of the cell wall, pectin is considered to be the main Cd binding molecular, owing to the strong ion exchange activity (Loix et al., 2017; Guo et al., 2021). The Cd ions (Cd^2+^) binding capacity of pectin requires the catalytic release of its free carboxyl groups (-COOH) group by PME (Wu et al., 2020). It has been widely demonstrated that higher PMEs expression levels and PMEs activity enhance Cd binding of cell walls and impede Cd^2+^ entry into cells (Wu et al., 2021a; Huang et al., 2022). In this study, roots of MYQZ under Cd treatment exhibited lower PMEI and higher PME expression levels and activity, and FTIR absorbance also revealed that root cell wall of MYQZ contained more pectin, which ultimately led to stronger fixation of Cd by the MYQZ root cell wall.

According to the studies of *Brassica chinensis* L. and *Vicia sativa*, lignin content was involved in the differences of Cd tolerance and Cd accumulation characteristics between high- and low-Cd cultivars (Rui et al., 2016; Wang et al., 2020). In *Brassica chinensis* L., approximately 14.1% of Cd was adsorbed by lignin in the high-Cd cultivar, while 14.5% of Cd was adsorbed by the lignin of the low-Cd cultivar (Wang et al., 2020). The lignin synthesis-related genes (*CADs and PODs*), lignin content as well as FTIR analyses of lignin (peaks at 1238 and 1157 cm^-1^) in MYQZ were significantly higher (*p*<0.05) than that of BXGZ in this study, which are consistent with our previous study (Shen et al., 2021), revealing that the distribution of Cd in the root cell wall is significantly higher in MYQZ than in BXGZ. Collectively, differences in lignin should also be an important factor in determining the Cd accumulation characteristics of eggplant.

### 4.2 MYQZ enhanced vacuole compartmentalization of Cd *via* sulfur metabolism and Cd transporters

Sulfur metabolites such as GSH and PCs containing thiol groups (-SH) are crucial for plant growth in response to environmental stress (Cui et al., 2020). Plants have been shown to minimize the toxicity of Cd by elevating the GSH content and promoting the complexation of GSH and Cd^2+^ with the help of GST (Zhang et al., 2021). In this study, the sulfate transporter (*SULTR1;3 and SULTR4;2*) as well as the enzymes (*APS1*, *APR2*, *RCS3*, *GST1* and *3*) involved in sulfur metabolism and chelation of Cd were with significantly higher (*p*<0.05) expression in MYQZ than in BXGZ, and the contents of GSH, GSSG and their precursor cysteine were much higher (*p*<0.05) in MYQZ under Cd stress. Although MYQZ is Cd intolerant in terms of the biomass results, the enhanced sulfur metabolism should contribute to the mitigation of Cd toxicity.IRT1 is an important metal transporter that contributes to Cd uptake by roots (Barberon et al., 2014). The overexpression and knockdown of *IRT1* have been revealed to cause dynamical Cd contents increases and decreases in *Brassica chinensis* and *Arabidopsis thaliana* (Connolly et al., 2002; Wu et al., 2021b). Here we observed that the expression of *IRT1* was significantly higher (*p*<0.05) in MYQZ than in BXGZ under Cd stress. In addition, ABC, CAX, and MTP are key transporters responsible for transporting Cd^2+^ from the cytoplasm to the vacuole, which should contribute to the retaining of Cd^2+^ in root vacuoles for the sake of eliminating the Cd translocation and accumulation in shoot (Yang et al., 2022). The DEGs analysis showed that *ABC*, *CAX*, and *MTPs* were also expressed at significantly higher levels (*p*<0.05) in MYQZ than in BXGZ. In this study, although the higher expression level of *IRT1* in MYQZ could certainly promote the Cd uptake, it was very likely that the presence of *ABCs*, *CAXs* and *MTPs* constrained Cd in the vacuole and reduced the translocation of Cd to the shoot.

### 4.3 Root development and transpiration promote Cd uptake and translocation in BXGZ

The root tip is the most active root region for Cd uptake (Lux et al., 2011). It was observed that Cd accumulation in rice was positively correlated with the number of root tips, with fewer root tips leading to less Cd accumulation (Huang et al., 2019). Comparative studies of high- and low-Cd cultivars in potato and hot pepper revealed that high-Cd cultivar possessed longer root length, more root tips, larger root surface area and root volume, which should contribute to its higher Cd uptake and translocation (Huang et al., 2015; Xin et al., 2017). Our previous study revealed that the Cd net uptake *via* root of BXGZ under low and high Cd treatment was were significantly higher than those of MYQZ (Shen et al., 2021). In this study, with the significantly higher expression of root development genes (*ARF19*, *GATA4*, *5*, *11* and *PIN5*), BXGZ showed a more developed root system of BXGZ under Cd treatment, indicting the root development of eggplant should be a key factor affecting the Cd net uptake *via* roots.

Transpiration pull is the main driving force for Cd translocation from roots to shoots in plants (Khanna et al., 2022). Studies in *Arabidopsis* and grapevine uncovered a positive correlation between *aquaporin* expression levels and plant transpiration (Pou et al., 2013; Macho-Rivero et al., 2018). Moreover, the density and aperture of stomata are also the factors affecting the transpiration of plants (Carins Murphy et al., 2014). In *Sedum alfredii*, ABA regulated the transpiration rate by depressing the expression of root aquaporin (*SaPIP*), stomatal density and size, resulting in reduced Cd translocation from roots to shoots (Tao et al., 2021). Studies demonstrated that *PCaP1* promoted the stomatal closure, while *CYCA2-1* contributed to the stomatal development, both of which can affect transpiration rate in plants (Nagata et al., 2016; Qu et al., 2018). The higher expression level of *aquaporin* and *CYCA2-1*, and stomatal density along with the higher transpiration rate, which inevitably lead to a stronger driving force for translocation of Cd to the shoot of BXGZ. Conversely, the higher expression of *PCaP1* and the corresponding lower density and transpiration rate would limit the transport of Cd to the shoot in MYQZ.

## 5 Conclusion

This study attempted to elucidate the mechanisms underlying the differences in Cd accumulation capacity between eggplant high-Cd cultivar (BXGZ) and low-Cd cultivar (MYQZ). On the one hand, MYQZ enhanced the fixation of Cd in the cell wall system by up-regulating the *PMEs*, *CADs* and *PODs* as well as enhancing pectin activity and lignin content in the root cell wall. Meanwhile, the Cd toxicity was also minimized by higher sulfur metabolism of thiol (SH)-containing products in MYQZ minimized Cd toxicity and reduced Cd translocation by compartmentalizing Cd within the vacuole through the higher expression of *ABCs*, *CAXs*, and *MTPs.* On the other hand, the uptake and translocation of Cd by BXGZ can be enhanced by the developed root system and stronger transpiration rate. Moreover, the relatively weak Cd fixation in the root of BXGZ should also be responsible for the easier translocation of to the shoot *via* the symplast and apoplast pathways.

## Data availability statement

The original contributions presented in the study are included in the article/**Supplementary Material**. The data presented in the study are deposited in the SRA repository, accession number PRJNA908752. Further inquiries can be directed to the corresponding author.

## Author contributions

CS and H-LF: Conceptualization, Methodology, Software. CS: Data curation, Writing- Original draft preparation. Y-YH and QL: Visualization, Investigation. H-LF, BH: Supervision. J-LX, LW: Software, Validation. CS and H-LF: Writing- Reviewing and Editing. All authors contributed to the article and approved the submitted version.
