# Supplementary material for: Characterization of cadmium accumulation mechanism between eggplant (Solanum melongena L.) cultivars
Source: Front Plant Sci. 2023 Jan 9;13:1097998. doi: 10.3389/fpls.2022.1097998 (PMC9868947; doi:10.3389/fpls.2022.1097998)
Supplement: Supplementary file 1 [file DataSheet_1.docx]

**Characterization of cadmium accumulation mechanism between eggplant (*Solanum melongena* L.) cultivars**

Chuang Shen, Ying-Ying Huang, Qiong Liao, Baifei Huang, Jun-Liang Xin, Luo Wang, Hui-Ling Fu *

Research Center for Environmental Pollution Control Technology, School of Chemical and Environmental Engineering, Hunan Institute of Technology, Hengyang 421002, China

* Corresponding Author

Hui-Ling Fu *

Mail address: Heng Hua Road 18, Hengyang, 421002, China.

Email: fuhl040816@hnit.edu.cn

Tel:+86-15622793497

**Tables**

Table S1 Two-way analysis of variance (ANOVA) for shoot biomass.

| Source of variance | Sum of squares | Mean square | F value | P value |
| --- | --- | --- | --- | --- |
| treatment | 0.012 | 0.012 | 27.254** | 0.001 |
| cultivar | 0.16 | 0.16 | 365.108** | 0 |
| treatment * cultivar | 1.14E-06 | 1.14E-06 | 0.003 | 0.961 |
| Error | 0.004 | 0 |  |  |
| Total | 2.456 |  |  |  |

Note: ** indicated significant difference at *p* < 0.01 level.

Table S2 Two-way analysis of variance (ANOVA) for root biomass.

| Source of variance | | Sum of squares | | Mean square | | F value | | P value | |  |  |
| --- | --- | --- | --- | --- | --- | --- | --- | --- | --- | --- | --- |
| treatment | 0.022 | | 0.022 | | 33.544** | | | 0 | | | |
| cultivar | 0.126 | | 0.126 | | 189.513** | | | 0 | | | |
| treatment * cultivar | 0.001 | | 0.001 | | 0.908 | | | 0.368 | | | |
| Error | 0.005 | | 0.001 | |  | |  | |  |  |  |
| Total | | 0.721 | |  | |  | |  | | |  |

Note: ** indicated significant difference at *p* < 0.01 level.

Table S3 Two-way analysis of variance (ANOVA) for shoot Cd concentration.

| Source of variance | Sum of squares | | | Mean square | | F value | P value | | |  |
| --- | --- | --- | --- | --- | --- | --- | --- | --- | --- | --- |
| treatment | | 61.255 | 61.255 | | 1499.775** | | | 0 |  |  |
| cultivar | | 2.248 | 2.248 | | 55.044** | | | 0 |  |  |
| treatment * cultivar | | 2.311 | 2.311 | | 56.58** | | | 0 |  |  |
| Error | | 0.327 | 0.041 | |  | | |  |  |  |
| Total | 128.285 | | |  | |  |  | | | |

Note: ** indicated significant difference at *p* < 0.01 level.

Table S4 Two-way analysis of variance (ANOVA) for root Cd concentration.

| Source of variance | Sum of squares | | Mean square | | | F value | | P value | |  |
| --- | --- | --- | --- | --- | --- | --- | --- | --- | --- | --- |
| treatment | | 3293.917 | | 3293.917 | 1560.795** | | 0 | |  |  |
| cultivar | | 24.254 | | 24.254 | 11.492** | | 0.009 | |  |  |
| treatment * cultivar | | 23.914 | | 23.914 | 11.331** | | 0.01 | |  |  |
| Error | | 16.883 | | 2.11 |  | |  | |  |  |
| Total | 6738.661 | |  | | |  | |  | | |

Note: ** indicated significant difference at *p* < 0.01 level.

Table S5 Two-way analysis of variance (ANOVA) for transpiration rate.

| Source of variance | Sum of squares | | Mean square | | | | F value | | | P value | |  |
| --- | --- | --- | --- | --- | --- | --- | --- | --- | --- | --- | --- | --- |
| treatment | | 224.468 | | 224.468 | | 66.591** | | | 0 | | | |
| cultivar | | 204.187 | | 204.187 | | 60.575** | | | 0 | | | |
| treatment * cultivar | | 5.741 | | 5.741 | | 1.703 | | | 0.228 | | | |
| Error | | 26.967 | | 3.371 |  | | |  | | |  |  |
| Total | | 6850.83 | |  |  | | |  | | |  |  |

Note: ** indicated significant difference at *p* < 0.01 level.

Table S6 Two-way analysis of variance (ANOVA) for number of root tips.

| Source of variance | Sum of squares | | | Mean square | | F value | | P value | |
| --- | --- | --- | --- | --- | --- | --- | --- | --- | --- |
| treatment | | 319154.083 | 319154.083 | | 122.878** | | 0 | |  |
| cultivar | | 539752.083 | 539752.083 | | 207.81** | | 0 | |  |
| treatment * cultivar | | 32344.083 | 32344.083 | | 12.453** | | 0.008 | |  |
| Error | | 20778.667 | 2597.333 | |  | |  | |  |
| Total | | 8648131 |  | |  | |  | |  |

Note: ** indicated significant difference at *p* < 0.01 level.

Table S7 Two-way analysis of variance (ANOVA) for number of root surface area.

| Source of variance | Sum of squares | | Mean square | | | F value | | P value | | |
| --- | --- | --- | --- | --- | --- | --- | --- | --- | --- | --- |
| treatment | | 870.403 | | 870.403 | 375.984** | | 0 | | |  |
| cultivar | | 1071.63 | | 1071.63 | 462.907** | | 0 | | |  |
| treatment * cultivar | | 9.363 | | 9.363 | 4.045 | | 0.079 | | |  |
| Error | | 18.52 | | 2.315 |  | |  | | |  |
| Total | | 14424.88 | |  |  | |  | |  |  |

Note: ** indicated significant difference at *p* < 0.01 level.

Table S8 Two-way analysis of variance (ANOVA) for lignin content.

| Source of variance | Sum of squares | | Mean square | | | F value | | P value |  |
| --- | --- | --- | --- | --- | --- | --- | --- | --- | --- |
| treatment | | 663.053 | | 663.053 | 53.699** | | 0 | | |
| cultivar | | 1456.403 | | 1456.403 | 117.951** | | 0 | | |
| treatment * cultivar | | 84.27 | | 84.27 | 6.825* | | 0.031 | | |
| Error | | 98.78 | | 12.347 |  | |  | | |
| Total | | 67541.76 | |  |  | |  | | |

Note: *and** indicated significant difference at *p* < 0.05 and *p* < 0.01 level, respectively.

Table S9 Two-way analysis of variance (ANOVA) for PME activity.

| Source of variance | Sum of squares | | Mean square | | | F value | P value | |
| --- | --- | --- | --- | --- | --- | --- | --- | --- |
| treatment | | 0.307 | | 0.307 | 30.593** | | 0.001 |  |
| cultivar | | 1.216 | | 1.216 | 121.099** | | 0 |  |
| treatment * cultivar | | 0.077 | | 0.077 | 7.648* | | 0.024 |  |
| Error | | 0.08 | | 0.01 |  | |  |  |
| Total | | 20.984 | |  |  | |  |  |

Note: *and** indicated significant difference at *p* < 0.05 and *p* < 0.01 level, respectively.

Table S10 Two-way analysis of variance (ANOVA) for cysteine content.

| Source of variance | Sum of squares | | Mean square | | | F value | | P value | |
| --- | --- | --- | --- | --- | --- | --- | --- | --- | --- |
| treatment | | 1.541 | | 1.541 | 4.905 | | 0.058 | |  |
| cultivar | | 15.188 | | 15.188 | 48.342** | | 0 | |  |
| treatment * cultivar | | 2.001 | | 2.001 | 6.369* | | 0.036 | |  |
| Error | | 2.513 | | 0.314 |  | |  | |  |
| Total | | 508.93 | |  |  | |  | |  |

Note: *and** indicated significant difference at *p* < 0.05 and *p* < 0.01 level, respectively.

Table S11 Two-way analysis of variance (ANOVA) for GSH content.

| Source of variance | Sum of squares | | Mean square | | | F value | | P value |  |
| --- | --- | --- | --- | --- | --- | --- | --- | --- | --- |
| treatment | | 9.72 | | 9.72 | 103.221** | | 0 | | |
| cultivar | | 4.563 | | 4.563 | 48.46** | | 0 | | |
| treatment * cultivar | | 0.003 | | 0.003 | 0.035 | | 0.855 | | |
| Error | | 0.753 | | 0.094 |  | |  | | |
| Total | | 123.04 | |  |  | |  | | |

Note: ** indicated significant difference at *p* < 0.01 level.

Table S12 Two-way analysis of variance (ANOVA) for GSSG content.

| Source of variance | Sum of squares | | Mean square | | | F value | | P value |  |
| --- | --- | --- | --- | --- | --- | --- | --- | --- | --- |
| treatment | | 6.092 | | 6.092 | 479.361** | | 0 | | |
| cultivar | | 1.062 | | 1.062 | 83.573** | | 0 | | |
| treatment * cultivar | | 1.122 | | 1.122 | 88.321** | | 0 | | |
| Error | | 0.102 | | 0.013 |  | |  | | |
| Total | | 24.735 | |  |  | |  | | |

Note: ** indicated significant difference at *p* < 0.01 level.

Table S13 Two-way analysis of variance (ANOVA) for POD activity.

| Source of variance | Sum of squares | | Mean square | | | | F value | | | P value | |
| --- | --- | --- | --- | --- | --- | --- | --- | --- | --- | --- | --- |
| treatment | | 1540.803 | | 1540.803 | | 74.902** | | | 0 | | |
| cultivar | | 5042.734 | | 5042.734 | | 245.14** | | | 0 | | |
| treatment * cultivar | | 118.755 | | 118.755 | | 5.773* | | | 0.043 | | |
| Error | | 164.566 | | 20.571 | |  | | |  | | |
| Total | | 117572.742 | |  |  | | |  | | |  |

Note: *and** indicated significant difference at *p* < 0.05 and *p* < 0.01 level, respectively.

**Figures**


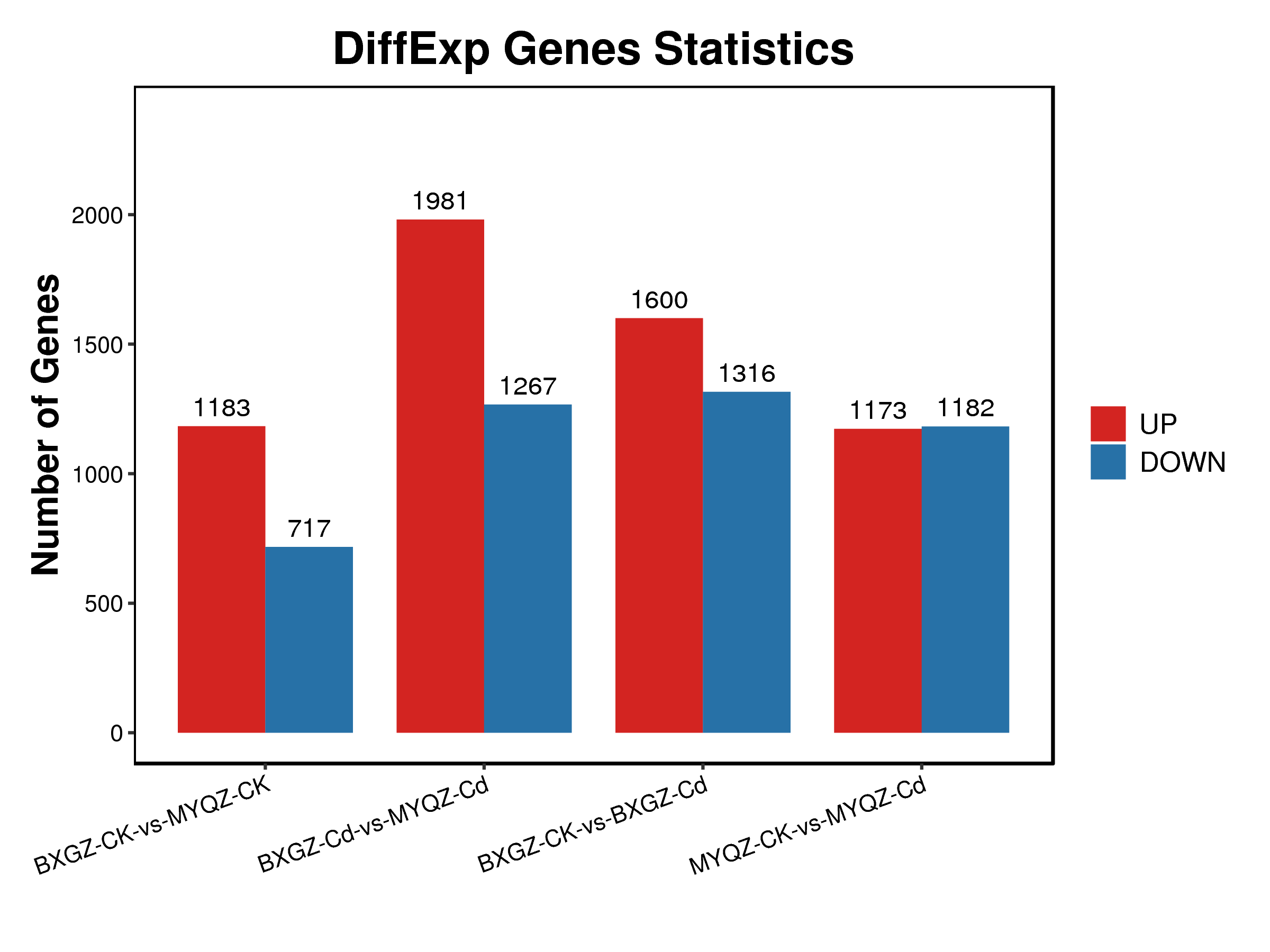


Figure S1. The number of DEGs among the four transcriptome profiles.


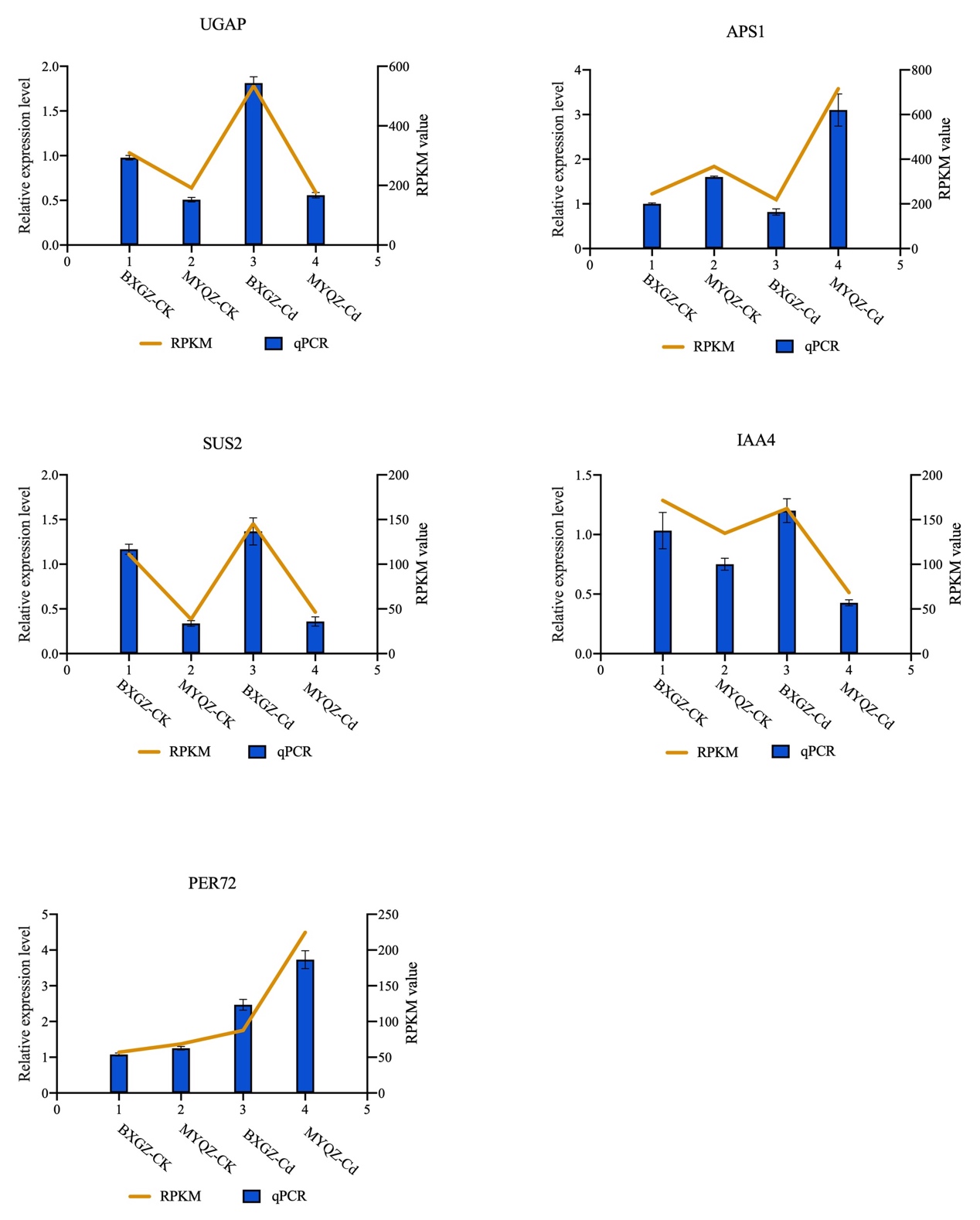


Figure S2. The expression of the selected 5 mRNAs inferred by mRNA sequencing. Note: error bars represent standard deviation (n=3).
