# Supplementary figures and images for: Characterization of cadmium accumulation mechanism between eggplant (Solanum melongena L.) cultivars
Source: Front Plant Sci. 2023 Jan 9;13:1097998. doi: 10.3389/fpls.2022.1097998 (PMC9868947; doi:10.3389/fpls.2022.1097998)

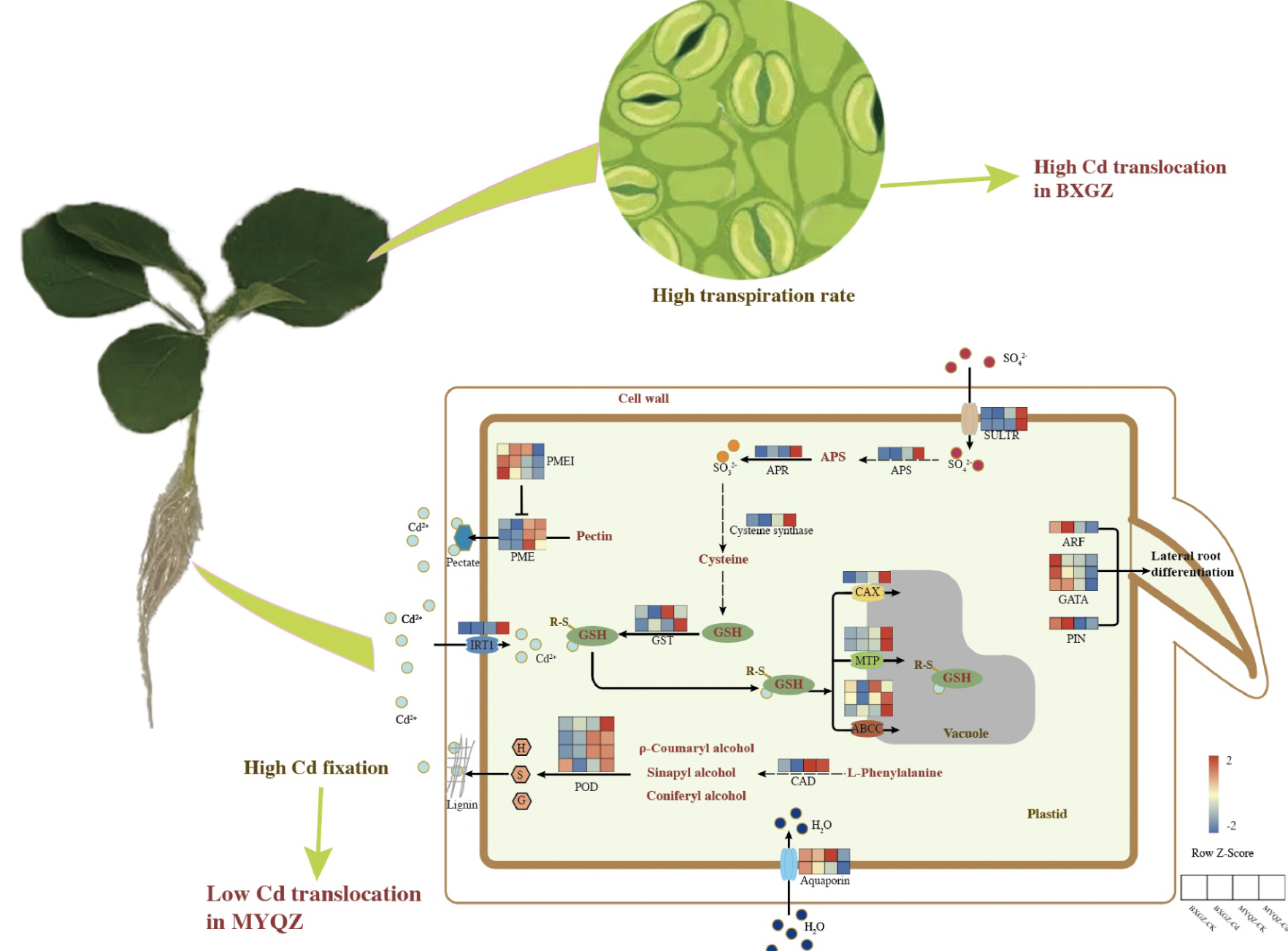

Supplement: Supplementary file 2 [file DataSheet_2.docx]
